# Adolescent Lifestyle Behaviors, Coping Strategies and Subjective Wellbeing during the COVID-19 Pandemic: An Online Student Survey

**DOI:** 10.3390/healthcare8040472

**Published:** 2020-11-09

**Authors:** Yolande Pigaiani, Leonardo Zoccante, Anastasia Zocca, Athos Arzenton, Marco Menegolli, Sabrina Fadel, Mirella Ruggeri, Marco Colizzi

**Affiliations:** 1 A.R.S.D.A. Association Research Study Alimentary Disorders, 37122 Verona, Italy; ypigaiani@tiscali.it (Y.P.); anastasia.zocca@studenti.unipd.it (A.Z.); marcomene87@gmail.com (M.M.); s.fadel0101@gmail.com (S.F.); 2 Child and Adolescent Neuropsychiatry Unit, Maternal-Child Integrated Care Department, Integrated University Hospital of Verona, 37126 Verona, Italy; leonardo.zoccante@aovr.veneto.it; 3 Istituto di Istruzione Superiore Ferraris-Fermi, 37122/37138 Verona, Italy; athos.arzenton@gmail.com; 4 Section of Psychiatry, Department of Neurosciences, Biomedicine and Movement Sciences, University of Verona, 37134 Verona, Italy; mirella.ruggeri@univr.it; 5 Department of Psychosis Studies, Institute of Psychiatry, Psychology and Neuroscience, King’s College London, London SE5 8AF, UK

**Keywords:** adolescence, resilience, stress, family, school, child and adolescent psychiatry, mental health prevention

## Abstract

Background and objectives: Adolescence represents a critical period for rapid psychophysical and socio-cognitive changes, with implications for health and wellbeing in later life. From this perspective, the manifestation of unhealthy lifestyles and dysfunctional behaviors may reflect a change in wellbeing requiring alertness and prompt intervention. This study investigated lifestyle behaviors and coping strategies among Italian adolescents, also in relation to the ongoing COVID-19 pandemic, and whether they would predict a change in subjective wellbeing. Materials and Methods: In the period between 1 April and 10 April 2020, adolescents aged 15–21 filled out an online survey consisting of 33 questions investigating socio-demographic characteristics, lifestyle behaviors, coping strategies, and subjective wellbeing. Results: Data was available on 306 participants. Most adolescents planned their daily routine (57.8%), engaging in structured activities (17.6–67.3%) and developing new interests (54.6%), and gave a positive reading of the ongoing period (57.8%), thus revealing adaptive coping strategies. Family wise, even though it was hard to stay at home (66%) and difficulties emerged, including self-isolation (50.7%) and quarrels (31.7%), a relevant proportion of adolescents shared their feelings (40.5%) and revaluated their family relationships (29.4–39.7%). In terms of social and school engagement, almost all adolescents kept contacts with their partner, friends, and teachers (90.2–93.5%). School commitments at home were sufficiently preserved (63.1%), however adolescents expressed preoccupations about their educational path (56.2%). A change in subjective wellbeing (49.3%) and symptoms of anxiety (39.9%) were frequently reported. A number of factors predicted a change in subjective wellbeing, including adaptive coping strategies (physical activity, OR = 2.609, 95% confidence interval (CI) 1.297–5.247; engaging in different activities than before, OR = 2.212, 95% CI 1.157–4.230), family issues (finding hard to stay at home, OR = 3.852, 95% CI 1.953–7.599; having quarrels, OR = 2.158, 95% CI 1.122–4.150), school-related behaviors (fearing a negative educational outcome, OR = 1.971, 95% 1.063–3.655), and female gender (OR = 3.647, 95% CI 1.694–7.851). Conclusions: Both personal and environmental coping resources are relevant to subjective wellbeing in adolescence and should be taken into account for prevention and early intervention in youth mental health.

## 1. Introduction

Adolescence represents a critical period for rapid physical, social, cognitive, and emotional changes, with important implications for health and wellbeing in later life [1]. Better healthcare and nutrition, extended education, and a widespread use of new technologies, offer the possibility of this being the healthiest generation of adolescents ever [2]. However, nowadays new threats to adolescents’ wellbeing have emerged, including wider availability of unhealthy products, the global youth unemployment crisis, poorer family stability, environmental deterioration, war conflicts, and mass migration [2]. Recent times have also witnessed the worldwide spread of a new viral pathogen, whose potentially deadly infectious disease, the 2019 coronavirus disease (COVID-19), may represent a stressful life event [3]. Regardless of historical period, consolidated evidence indicates that most mental health difficulties have their peak of incidence during the transition from childhood to young adulthood, affecting up to 20% of adolescents [4]. Moreover, among people younger than 25 years old, psychological and emotional difficulties account for health loss in almost one case in two [5]. From this perspective, one of the areas requiring alertness and prompt intervention is the manifestation of unhealthy lifestyles and dysfunctional behaviors, a pattern that increases with age, independent of its clinical relevance [6]. On the other hand, acquiring social-emotional skills such as self-control, self-regulation, and goal-directed behavior predicts better academic and social adjustment as well as overall youth wellbeing [7,8]. 

Skill development during adolescence takes place within a complex social network made by family, peers, school, community, media, and other cultural influences. First, up to adolescence, families and related social conditions exert a strong influence on children’s wellbeing and competencies, with long-lasting effects throughout their lifespan [9]. Second, the school system, besides sustaining adolescents through their educational objectives and verifying their learning outcomes, is a platform for wellbeing promotion. Its mentoring and social support advocacy [10] provides not only basic health knowledge (e.g., notions of sex education) but also helps developing and maintaining lifestyles that minimize health risks [2]. Third, participation and civic engagement are important aspects of adolescents’ social development and personal fulfillment [1], as they engage progressively more with the wider social environment in search of a sense of community. The latter will require a new balance in the adolescent’s parent–child relationship and impact on their social behaviors and wellbeing [11]. Fourth, a considerable part of the adolescents’ development will happen in virtual space, as the last 30 years have seen the growing role of the internet as an instrument to create and maintain interpersonal relationships, whose opportunities and risks are constantly debated [12]. Fifth, the acquisition of health-related skills and resources would lay the foundations not only for the physical and mental wellbeing of the adolescents themselves, positively shaping their health trajectory throughout their lifespan, but also of the next generation, whom they will be parenting [13].

Altogether, evidence on the importance of sustaining health trajectories during adolescence has fueled a pro-active and positive interpretation of the wellbeing concept, understood as the progressive development of physical, emotional, and cognitive skills and not as the bare absence of negative attitudes [2]. To fully implement such change in the way adolescents’ wellbeing is considered, a shift towards a more pro-active and positive approach is also required for the wider youth-oriented mental health promotion and prevention strategies [14]. For instance, in order to support adolescents to live healthy lives, a broad range of preventive interventions are required, including self-care. Self-care is particularly relevant during adolescence when the concept of self is fully developing and consolidating. It allows adolescents and their families to sustain their health through health-promoting practices, monitoring of one’s own changes in habits and behaviors, and responding to occurring health threats, also at the cognitive and emotional level [15]. In fact, as for physical wellbeing, self-evaluation is crucial to promote mental and social wellbeing, as parts of the individual’s overall subjective wellbeing [16]. Consistent, subjective wellbeing during adolescence has been suggested to be intimately related with good self-assessment and knowledge of one’s state of health [17], positive attitudes towards life [17], school satisfaction [17,18], social support [7], positive social climate within family [19], engagement with healthy behaviors (e.g., physical activity) [17,20], and avoidance of unhealthy habits (e.g., smoking) [17].

The purpose of this study was threefold. The main aim was to rapidly investigate lifestyle behaviors, coping strategies, and subjective wellbeing among adolescent students. Following from previous evidence [17,18,19], a further aim was to conduct exploratory analyses to investigate whether any sociodemographic characteristics, lifestyle behaviors or coping strategies would be associated with a change in subjective wellbeing. Finally, the survey served to characterize how adolescents’ subjective wellbeing changed in relation to the ongoing COVID-19 pandemic.

## 2. Materials and Methods

### 2.1. Research Design

An online student survey was created by using Google Forms and then disseminated via a hyperlink, within the educational digital platform already adopted by each school to support management processes and to improve record-keeping of the educational process. The survey was available online from the 1 April to the 10 April 2020. All students aged 18 years and older, or parents for those students less than 18 years old, provided electronic informed consent containing information about the purpose of the study, procedures, benefits of participating, voluntary participation, and contact information of the researchers.

The survey was part of a larger study which was approved by the research ethics committee at the Integrated University Hospital of Verona (CESC 2242).

### 2.2. Participants

A nonprofit organization, with a mission to empower wellbeing by educating healthcare professionals, school personnel, advocacy and support networks about preventative health, as well as school networks with a mission to empower economic, social, cultural, civic and personal wellbeing among students by the implementation of Information and Communication Technologies and digital inclusion, were used to distribute and directly encourage survey participation. The online student survey was proposed to three Italian high schools throughout the country, which were already collaborating with the nonprofit organization and school networks. Through the involvement of the School Governing Bodies, students were asked to fill out the online survey.

### 2.3. Instrument

The student survey was developed by a focus group of physicians, psychologists, and child life specialists, also taking advice from the nonprofit organization and school networks. The survey consisted of 3 socio-demographic questions and 30 questions exploring lifestyle behaviors, coping strategies, and subjective wellbeing (29 multiple choice questions, 1 yes/no question). Participants were allowed to select only 1 item for each question.

### 2.4. Analyses

The final raw data was downloaded from Google Forms into a Microsoft Excel file for analysis using SPSS software (Version 26.0; IBM Corp, Armonk, NY, USA). Descriptive statistics were used to provide baseline information concerning students’ socio-demographic characteristics, lifestyle behaviors, coping strategies, and subjective wellbeing. In order to gain interpretability and simplicity as well as take into account potentially highly skewed and/or binomial distributions, when applicable, multiple response options were also restructured into dichotomous variables by collapsing data points into high (enough/very; often/always) and low (not at all/a little; never/seldom) groups. Then, a multiple logistic regression was performed to investigate whether any students’ socio-demographic characteristics, lifestyle behaviors, or coping strategies would predict a change in their subjective wellbeing.

The survey was not intended to formally conduct mental health assessments of participating students.

## 3. Results

### 3.1. Sociodemographic Characteristics

A total of 306 adolescents participated in the survey, coming from three high schools located in Verona (56.5%), Milan (15.7%), and Rome (27.8%), Italy. The mean age of participating students was 18.1 years (SD = 0.9; range: 15–21). Male students represented 72.9% of the sample.

A full description of survey responses, including all multiple-choice options, is provided in Table 1 and Table 2. The following data reporting describes adolescent students based on the dichotomization of the sample. In particular, it refers to the high group’s behavioral patterns (enough/very; often/always).

### 3.2. Lifestyle Behaviors and Coping Strategies

Following the COVID-19 emergency outbreak, an overwhelming proportion of students put an effort to respect the rules in place in everyone’s best interest (95.1%). Most students planned their daily routine (57.8%), also deciding to organize their time in a different way (82%) and to engage in different activities (54.6%) than before. Most common activities were doing physical activity (67.3%), getting interested in cooking (47.4%), playing videogames (36.3%), reading (30.1%), and playing board games (17.6%).

Even though one in every two students tended to frequently withdraw into their bedroom (50.7%), with one third reporting a significant increase in family quarrels (31.7%), a substantial proportion of adolescents let their parents know their feelings (40.5%) and a slightly lower percentage revaluated their parent–child (29.4%) or sibling relationships (39.7%). Of those having a romantic relationship, the large majority reported missing their partner and supporting each other (90.2%). Almost all students used social networks to keep contact with their friends (93.5%) and some of them made new acquaintances online (19.3%). Asked to choose between four descriptions of the ongoing period, the positive ones, that are “useful–opportunity” (30.4%) and “support–nearness” (27.4%), were the most selected.

Apart from a few students, all kept remote contacts with their teachers (92.2%). Most adolescents considered positively the offered distance learning (79.1%), reporting being able to do their homework (63.1%) and to balance school commitments and free time (63.1%). Personal computers were the most commonly used devices for distance learning (55.2%). However, more than half of the students feared a negative impact of the ongoing period on their educational path (56.2%) (Table 1).

### 3.3. Subjective Wellbeing

For most students, it was hard to stay at home (66%). One in two students felt considerably changed by the experience of the pandemic (50.7%) and noticed a significant change in their psychological wellbeing (49.3%). More than one third reported being particularly anxious about the ongoing situation (39.9%). Even though three out of four students reported going to bed late often or always (81%), most of them self-evaluated their sleep–waking cycle as generally preserved (59.2%) (Table 2).

### 3.4. Predictors of Change in Subjective Wellbeing

A multiple logistic regression tested for an effect of the investigated socio-demographic characteristics, lifestyle behaviors, and coping strategies on the subjective change in psychological wellbeing. The logistic regression model was statistically significant, χ2 (28, N = 303) = 125.935, *p* < 0.001. The model explained 45.3% (Nagelkerke R2) of the variance and correctly classified 76.9% of cases.

Students who found it hard to stay at home (OR = 3.852, 95% confidence interval (CI) 1.953–7.599), argued more easily with family members (OR = 2.158, 95% CI 1.122–4.150), and feared a negative impact of the ongoing period on their educational path (OR = 1.971, 95% CI 1.063–3.655), were all more likely to report a subjective change in their psychological wellbeing. Interestingly, being female (OR = 3.647, 95% CI 1.694–7.851), doing physical activity (OR = 2.609, 95% CI 1.297–5.247), and engaging in different activities than before (OR = 2.212, 95% CI 1.157–4.230), were all associated with a higher likelihood of reporting a subjective change in one’s psychological wellbeing. Furthermore, reevaluating the parent–child relationship and feeling anxious about the ongoing situation were both weakly associated with a change in one’s psychological wellbeing.

Instead, students describing the ongoing period with positive words (e.g., Useful—opportunity; Support—nearness) were less likely to report a subjective change in wellbeing (OR = 0.377, 95% CI 0.194–0.735). Also, being able to work at home and do personal homework quietly was weakly associated with a lower likelihood of reporting a subjective change in one’s psychological wellbeing (Table 3).

## 4. Discussion

The current study aimed to extend prior research on lifestyle behaviors and coping strategies during the adolescent period, also focusing on how such mechanisms can predict adolescents’ subjective well-being in general and at times of potential psychosocial crisis as the COVID-19 emergency outbreak in particular. Results from this survey suggest a number of “active” and planning” adaptive coping strategies. In particular, most adolescents reported being organized and purposeful in their use of time, spending it in structured activities and developing new interests, and gave a positive reading of the ongoing period. In terms of family support, on one hand it was certainly hard to stay at home for most adolescent students and difficulties in family functioning emerged, with self-isolation and quarrels being frequently reported. However, despite such maladaptive coping styles, on the other hand a relevant proportion of adolescents shared their feelings and revaluated their family relationships. In terms of social support, almost all adolescents remained in contact with their partner and friends, supporting each other. With reference to school engagement, almost all adolescents had contacts with their teachers, however school commitments at home appeared to be only sufficiently preserved, and a large proportion of students expressed preoccupations about their educational path. Regarding their subjective wellbeing, about one in two adolescents experienced a relevant change and one in three reported symptoms of anxiety.

When assessing the impact of adolescents’ lifestyle behaviors and coping strategies on their psychological wellbeing, a number of variables predicted a significant change. “Active” and planning” adaptive coping strategies associated with a significant change in wellbeing included doing physical activity and engaging in different activities than before. This is consistent with evidence that self-regulation, choice, active response, and volition are adaptive to performance [21,22]. Self-control enables children and adolescents to cope with the experience of emotional challenges or difficult situations [23].

Family-related behaviors predicting a change in wellbeing included finding it hard to stay at home, having quarrels, and, at a trend level, reevaluating the parent–child relationship. Regarding the potential impact of family difficulties on the ability of adolescents to sustain their own wellbeing, evidence on whether the manifestation of higher negative affect would also be associated with decreased positive affect is far from unequivocal [24]. Adolescents may respond to family difficulties in various ways, depending on the activation of this or that coping strategy [25].

At the school level, fearing a negative educational outcome predicted a subjective change in the psychological wellbeing. Instead, at a trend level, adolescents being able to quietly study at home, were less likely to perceive a change in their wellbeing. These findings extend previous evidence that higher school demands are associated with more perceived stress while schools can promote student wellbeing by providing clear and timely information and teacher support to the students [26].

Furthermore, male and female adolescents differed in self-report wellbeing, with females reporting a subjective change more likely. This is in line with previous evidence that females tend to assess events as more severe than males, possibly because of their ability to express a larger range of stronger and more complex emotions [27,28,29]. Also, with reference to the perception of the ongoing period, feeling anxious about it was weakly associated with a change in wellbeing, while those adolescents describing it positively were less likely to perceive a change in their wellbeing.

Surveys conducted during past infectious diseases with pandemic potential have allowed to collect timely data to provide evidence-based response strategies [30]. Consistent, the current online survey turned out to be a successful data gathering solution, whose advantages were the possibility to study a sufficiently large sample and expedite response times and data processing, also reducing costs [31]. Also, thanks to the support of the nonprofit organization and school networks as well as the involvement of School Governing Bodies, we limited the risk of noncontact or refusals that can reduce the response rate and representativeness of the sample, gathering data from more than three hundred adolescents in three different Italian regions. Nevertheless, results of this survey need to be interpreted in light of its limitations. Specifically, despite the previously mentioned strengths, coping strategies and subjective wellbeing were not assessed using standardized instruments, limiting the possibility to compare results between different studies or data collected at different time points from the same subjects.

## 5. Conclusions

Subjective wellbeing represents a major life goal and is crucial to optimal flourishing and functioning at the psychological, physical, and interpersonal level [32]. Consistent with this, it is used as a key outcome in several fields of psychology and mental health [33]. Even though most studies examining subjective wellbeing have traditionally focused on adult populations, recent years have witnessed an upsurge of interest in the role of subjective wellbeing for adolescent health and development [34,35,36,37,38]. In particular, during normative adolescence, attaining high levels of subjective wellbeing may reflect a successful and constructive psychosocial adjustment to one of the most rapid phases of human development [39]. Achieving subjective wellbeing is of even greater importance when adolescents additionally experience a stressful life event, due to its critical role in coping with the demands of the stressful situation, becoming resilient with respect to the negative event, and thus avoiding a functional impairment [40]. Resilient adolescents, who show the ability to recover from negative experiences, have been suggested to adapt more easily to the ever-changing demands of their transition age and to be less susceptible to the impact of unhealthy lifestyle behaviors on wellbeing [41]. Such evidence has important implications for the investigation of risk factors [42] as well as the development of effective early intervention and prevention strategies for mental health difficulties in youth [14].

In sum, this study focused on adolescents’ subjective wellbeing as a function of their lifestyle behaviors and coping strategies. Evidence from this study indicates that with regard to the role played by developmental factors, adolescents’ age did not predict any change in subjective well-being, whereas gender did, with females reporting such a change more likely. Also, both personal and environmental coping resources resulted to be relevant to subjective wellbeing in adolescence, including pursuing different activities such as physical activity on one hand, and receiving family as well as school support on the other.

## Figures and Tables

**Table 1:** Lifestyle behaviors and coping strategies.

Survey Questions	N	%
I put an effort to respect the rules in place in everyone’s best interest		
Very	195	63.7
Enough	96	31.4
A little	13	4.2
Not at all	2	0.7
I plan my daily routine		
Very	48	15.7
Enough	129	42.2
A little	101	33
Not at all	28	9.2
I decide to organize my time in a different way		
Very	92	30.1
Enough	159	52
A little	46	15
Not at all	9	2.9
I got engaged in different activities than before		
Very	38	12.4
Enough	129	42.2
A little	102	33.3
Not at all	37	12.1
I do physical activity		
Very	83	27.1
Enough	123	40.2
A little	64	20.9
Not at all	36	11.8
I got interested in cooking		
Very	38	12.4
Enough	107	35
A little	88	28.8
Not at all	73	23.9
I play videogames		
Very	42	13.7
Enough	69	22.5
A little	97	31.7
Not at all	98	32
I read		
Very	21	6.9
Enough	71	23.2
A little	119	38.9
Not at all	95	31
I play board games		
Very	8	2.6
Enough	46	15
A little	129	42.2
Not at all	123	40.2
I withdraw into my bedroom		
Very	65	21.2
Enough	90	29.4
A little	98	32
Not at all	53	17.3
I argue more easily with my family		
Very	21	6.9
Enough	76	24.8
A little	113	36.9
Not at all	96	31.4
I share my feelings with my family		
Very	25	8.2
Enough	99	32.4
A little	104	34
Not at all	78	25.5
I’ve been revaluating my relationship with my parents		
Very	15	4.9
Enough	75	24.5
A little	120	39.2
Not at all	96	31.4
I’ve been revaluating my relationship with my siblings		
Very	17	6.5
Enough	87	33.2
A little	86	32.8
Not at all	72	27.5
I’m only child	44	
If I have a partner, how much I miss him/her and support each other		
Very	126	58.6
Enough	68	31.6
A little	16	7.4
Not at all	5	2.3
I’m single	91	
I use social networks to keep in contact with my friends		
Very	181	59.2
Enough	105	34.3
A little	18	5.9
Not at all	2	0.7
I’m making new acquaintances on social networks		
Very	22	7.2
Enough	37	12.1
A little	99	32.4
Not at all	148	48.4
Choose which couple of words best describes the ongoing period		
Useful—opportunity	92	30.4
Support—nearness	83	27.4
Useless—frustration	54	17.8
Conflict—tension	74	24.4
Missing	3	
I maintain remote contacts with my teachers		
Very	123	40.2
Enough	159	52
A little	23	7.5
Not at all	1	0.3
How do I evaluate the offered distance learning in my school		
Exhaustive	61	19.9
Sufficiently adequate	181	59.2
Not very exhaustive	43	14.1
Poor	16	5.2
Inadequate	5	1.6
I am able to work at home and do my homework quietly		
Yes	193	63.1
No	113	36.9
I am able to balance school commitments and free time		
Very	46	15
Enough	147	48
A little	81	26.5
Not at all	32	10.5
Which device do I use most frequently for distance learning		
Notebook	29	9.5
PC Desktop	169	55.2
Smartphone	84	27.5
Tablet	24	7.8
I think that this period will affect my educational path		
Yes. in a positive way	66	21.6
Yes. in a negative way	172	56.2
No. it’s all the same	68	22.2

**Table 2:** Subjective wellbeing.

Items	N	%
How hard do I find to stay at home		
Very	120	39.2
Enough	82	26.8
A little	73	23.9
Not at all	31	10.1
I think this experience changed myself		
Very	28	9.2
Enough	127	41.5
A little	105	34.3
Not at all	46	15
I noticed significant changes in my psychological wellbeing		
Very	55	18
Enough	96	31.4
A little	92	30.1
Not at all	63	20.6
I feel anxious about the ongoing situation		
Very	37	12.1
Enough	85	27.8
A little	115	37.6
Not at all	69	22.5
I go to bed late		
Always	109	35.6
Often	139	45.4
Seldom	46	15
Never	12	3.9
I am able to preserve a balanced sleep-waking cycle		
Always	63	20.6
Often	118	38.6
Seldom	88	28.8
Never	37	12.1

**Table 3:** Predictors of change in subjective wellbeing.

	B	S.E.	Wald Chi-Square	*p*-Value	OR	95% C.I.
Gender (female)	1.294	0.391	10.937	0.001	3.647	1.694–7.851
Age	−0.155	0.170	0.833	0.361	0.856	0.614–1.195
I put an effort to respect the rules in place	0.335	0.659	0.258	0.611	1.398	0.384–5.089
I plan my daily routine	−0.062	0.352	0.031	0.861	0.940	0.471–1.876
I decide to organize my time in a different way	−0.058	0.401	0.021	0.885	0.943	0.430–2.072
I got engaged in different activities than before	0.794	0.331	5.766	0.016	2.212	1.157–4.230
I do physical activity	0.959	0.356	7.237	0.007	2.609	1.297–5.247
I got interested in cooking	−0.164	0.313	0.274	0.600	0.849	0.459–1.569
I play videogames	0.189	0.328	0.331	0.565	1.208	0.635–2.297
I read	−0.222	0.357	0.387	0.534	0.801	0.398–1.611
I play board games	−0.554	0.436	1.617	0.204	0.575	0.245–1.350
I withdraw into my bedroom	0.367	0.319	1.324	0.250	1.444	0.772–2.699
I argue more easily with my family	0.769	0.334	5.312	0.021	2.158	1.122–4.150
I share my feelings with my family	−0.392	0.351	1.248	0.264	0.676	0.340–1.344
I have been revaluating my relationship with my parents	0.601	0.358	2.827	0.093	1.824	0.905–3.676
I use social networks to keep in contact with my friends	1.030	0.689	2.236	0.135	2.802	0.726–10.812
I am making new acquaintances on social networks	−0.087	0.376	0.053	0.817	0.917	0.439–1.915
Words best describing the ongoing period (positive)	−0.975	0.340	8.220	0.004	0.377	0.194–0.735
I maintain remote contacts with my teachers	0.092	0.564	0.027	0.870	1.097	0.363–3.313
I evaluate the offered distance learning positively	0.475	0.376	1.592	0.207	1.608	0.769–3.362
I am able to work at home and do homework quietly	−0.556	0.337	2.727	0.099	0.574	0.297–1.109
I am able to balance school commitments and free time	−0.023	0.351	0.004	0.948	0.977	0.491–1.944
This period will negatively affect my educational path	0.678	0.315	4.635	0.031	1.971	1.063–3.655
I find hard to stay at home	1.349	0.347	15.144	<0.001	3.852	1.953–7.599
I think this experience changed myself	0.421	0.328	1.649	0.199	1.523	0.801–2.894
I feel anxious about the ongoing situation	0.581	0.342	2.881	0.090	1.787	0.914–3.493
I go to bed late	0.339	0.428	0.628	0.428	1.404	0.607–3.247
I am able to preserve a balanced sleep-waking cycle	−0.493	0.333	2.190	0.139	0.611	0.318–1.173
